# Prevalence of Periodontal Disease among Patients at the Outpatient Clinic of Internal Medicine in an Academic Hospital in The Netherlands: A Cross-Sectional Pilot Study

**DOI:** 10.3390/jcm11206018

**Published:** 2022-10-12

**Authors:** Thomas J. T. Leung, Nina Nijland, Victor E. A. Gerdes, Bruno G. Loos

**Affiliations:** 1 Department of Periodontology, Academic Center for Dentistry Amsterdam (ACTA), University of Amsterdam and Vrije Universiteit Amsterdam, Gustav Mahlerlaan 3004, 1081 LA Amsterdam, The Netherlands; 2 Department of Internal Medicine, Amsterdam University Medical Center (AUMC), 1105 AZ Amsterdam, The Netherlands; 3 Department of Internal Medicine, Spaarne Gasthuis, 2134 TM Hoofddorp, The Netherlands

**Keywords:** internal medicine, systemic disease, multimorbidity, cardiovascular disease, periodontal disease, oral health

## Abstract

There is a worldwide increase in individuals suffering ≥2 chronic diseases (multimorbidity), and the cause of combinations of conditions remains largely unclear. This pilot study analysed the prevalence of periodontal disease (PD) among (multi)-morbid patients at the outpatient clinic of internal medicine. PD is an inflammatory disease of the tooth supporting tissues and has a negative impact on the overall health. Data were obtained from 345 patients, on demographics, systemic conditions and presence of PD. The possible differences in the distribution of PD status among patients with/without multimorbidity and Medical Subject Headings (MeSH) disease chapters were explored. In total, 180 (52.2%) patients suffered from multimorbidity. The prevalence of severe PD was 16.2%, while the prevalence of mild and severe PD combined (Total PD) was 53.6%. Patients with disease chapter cardiovascular diseases (CVD) had a significantly higher prevalence of severe PD (odds ratio (OR) 2.33; 95% confidence interval (CI) 1.25, 4.33) and Total PD (OR 1.61; 95% CI 1.04, 2.50) than patients without CVD. After subsequent analyses, myocardial infarction was significantly associated with severe PD (OR: 4.68 (95% CI; 1.27 to 17.25)). Those suffering from multimorbidity showed to have a non-significant increased risk for severe (OR 1.27; 95% CI 0.69, 2.34) or Total PD (OR 1.23; 95% CI 0.81, 1.88). In conclusion, PD is highly prevalent in multimorbidity patients. Furthermore, PD was significantly prevalent in patients with CVD. However, larger epidemiological studies are necessary to confirm that the prevalence of PD is significantly increased among multimorbid patients.

## 1. Introduction

Non-communicable diseases (NCDs) are the main determinants of the global burden of diseases. NCDs are usually of long duration and are the leading cause of death, killing around 41 million people annually, accounting for 71% of global deaths [1]. NCDs include conditions such as atherosclerotic cardiovascular diseases (ACVD), diabetes mellitus (DM), cancers and chronic respiratory diseases. Currently, in several European countries, an increased prevalence is evident in individuals over 50 years suffering from two or more chronic conditions, including NCDs (multimorbidity) [2,3,4]. In individuals suffering from multimorbidity, certain NCDs can be causal to each other and/or share underlying disease mechanisms [5,6,7,8]. In that respect, pleiotropy for NCDs has been described as well [9]. However, the cause of multimorbidity remains largely unclear since certain potential relationships among these comorbidities remain unknown.

At the outpatient clinic for internal medicine, many patients suffer a minimum of one chronic disease, and multimorbidity is common. However, to date, no specific attention to oral conditions has been given. It is interesting to know what impact oral conditions may have on the overall health because oral conditions such as dental caries (tooth decay), periodontal (gum) disease and oral cancer are chronic, progressive in nature and share common risk factors with other NCDs (sugar intake, alcohol use, tobacco use, dysbiotic microbiomes, genetic risk factors and social determinants) [10,11]. Oral conditions, in particular periodontal disease (PD) (i.e., periodontitis), have been associated with systemic diseases and this phenomenon has become more evident in the last three decades [12,13,14,15].

PD is one of the most common inflammatory NCDs and it is reported to be the sixth most prevalent condition in the world with at least 10% of the global adult population suffering from the severe form [11,16]. PD leads to progressive destruction of tooth-supporting tissues and alveolar bone and, if left untreated, results in irreversible periodontal attachment loss, alveolar bone destruction, tooth mobility and eventually tooth loss [17,18,19]. This chronic disease not only has a negative impact on oral health and function, but there is growing evidence that PD may impact overall health via systemic inflammation and short-lived bacteraemias. Dental epidemiological studies investigating periodontitis patients and healthy controls, found strong evidence that PD is associated with some common NCDs, such as DM, ACVD and rheumatoid arthritis [12,13,14,15,20]. DM can be a cofactor in the aggravation of periodontitis in which hyperglycaemia in DM patient plays a role in the exacerbation of periodontitis [21]. Furthermore, it has been shown that severe periodontitis is frequently associated frequently ACVD [15,22,23,24]. In a large cohort study, an independent association between periodontitis and ACVD (including myocardial infarction, stroke and angina pectoris) was ascertained (odds ratio: 1.58) [13]. Interestingly, interventional studies in periodontitis patients with DM or ACVD portrayed that these comorbid conditions may improve when periodontal therapy is applied. In ACVD patients, improvement in endothelial function and a reduction in biomarkers were evident, while, in DM patients, beneficial metabolic control and a reduction in systemic inflammation and hemoglobin A1c (HbA1c) levels were observed [25,26].

Despite the described associations, the prevalence of PD among patients with NCDs or chronic infectious diseases is poorly investigated. PD was hypothesized to be associated with 57 systemic conditions, the majority being NCDs [27]. It can be suggested that periodontitis is part of a group of inflammatory diseases and that the mouth–body link is not only a consequence of common required risk factors, such as tobacco use and metabolic disbalance, but may also be driven by inflammatory mechanisms and pathways, such as microbial infection (bacteraemia and endotoxemia), long-standing low-grade inflammation (cytokinaemia) and intestinal dysbiosis (disruption of gut microbiota due to oro-pharyngeal or oro-digestive translocation of periodontal bacteria or pro-inflammatory mediators) [28]. These phenomena can initiate an inflammatory process in a distant organ or aggravate an existing chronic inflammatory lesion and, as such, could increase the severity of patients suffering from PD. This may explain the co-occurrence of PD and other systemic conditions. The current PD classification system is characterized by a multi-dimensional staging and grading classification system and includes systemic diseases. The presence of evident risk factors, such as smoking and diabetes (glycated hemoglobin A1c level), are considered and included in the grading process, and could modify the established grade, which is initially based on the evidence of progression [29]. Patients with PD due to specific rare syndromes, such as Papillon–Lefèvre Syndrome, are classified as having “Periodontitis as a Manifestation of Systemic Diseases” [30]. 

Many previous studies investigated the association between periodontitis and a specific systemic condition. However, very little research investigated whether PD is part of multimorbidity and disease phenotypes among patients in outpatient hospital clinics. The current research is a pilot study to analyse the possible presence of PD in multimorbid patients. Therefore, this present cross-sectional study aimed to assess the prevalence of PD among individuals visiting the outpatient clinic of internal medicine having one or more chronic condition(s).

## 2. Materials and Methods

### 2.1. Study Design

This was a cross-sectional pilot study consisting of secondary analyses based on the data from the studies from Nijland et al. (2021) [31] and Nijland et al. (2022) [32]. Therefore, no power-analysis or sample-size calculation took place. Patients were recruited from the outpatient clinic of the Department Internal Medicine at Amsterdam University Medical Center (AUMC). On specific research days, patients were approached at random. In total, there were 345 patients, based on two cohorts. The first cohort had 159 patients, recruited in 2019–2020 [31], and the second patient cohort comprised 186 patients, recruited in 2021–2022 during a follow-up study (Nijland et al. (2022) [32]). The main purpose of the Nijland et al. studies was to validate a self-reported questionnaire to screen for PD. For that purpose, oral examinations were also performed for the diagnosis of PD. One week prior to their scheduled visit to the outpatient clinic, the medical patients received an informative letter about the study. On arrival, the willing participants signed their informed consent form, and the pre-agreed procedure took place. Care was taken to exclude any previously participating patients. Patients from ≥18 and ≤90 years of age with at least one natural tooth present were able to participate. Patients <18 and >90 years of age and edentulous patients with or without full dentures (regardless of dental implant support) were excluded.

### 2.2. Data Collection 

The demographic data age, sex, educational qualifications (less or equal to high school or beyond) and ethnicity (Caucasian, non-Caucasian) were collected. Then, a clinical PD screening took place. Finally, the medical data per patient were extracted consisting of body mass index (BMI) and diseases (NCDs and chronic infectious diseases) from the AUMC medical data base (EPIC). 

The diseases from these patients were categorized by disease chapter using the 2022 Medical Subject Headings thesaurus (MeSH) from the U.S. National Library of Medicine. The MeSH thesaurus is a controlled and hierarchically organized vocabulary and is divided into 16 categories (branches), from A to N, V and Z. The (diseases) C and F branches were used to categorize the extracted diseases. According to the prevalence of the disease, participants were classified regarding multimorbidity: two or more systemic conditions are defined as multimorbidity [5]. All data per patient were entered into one secured online database (CASTOR EDC, Amsterdam, The Netherlands). A key document connected the research number with the corresponding patient number. A folder containing all documents was stored in a closed closet at the Department of Periodontology at ACTA.

### 2.3. Periodontal Disease Screening

Screening for PD was performed at the outpatient clinic of the Department of Internal Medicine. Due to the limited time available per patient, full-mouth periodontal examination was not possible. The periodontal condition of each patient was screened by applying the Community Periodontal Index of Treatment Needs (CPITN) [33,34]. The deepest measured pocket and bleeding on probing per sextant were used to score the CPITN to be either 0–2, 3 or 4. Those patients with CPITN-scores 0–2 in all sextants were categorized with no PD. Patients with at least one sextant with CPITN-score 3 but no CPITN-score 4, were categorized with ‘mild’ PD. If at least one sextant showed CPITN-score 4, the patient was categorized with ‘severe’ PD. For the analyses, we compared morbidities for (i) the patient group with severe PD with the rest or (ii) the patients with either mild or severe PD (combined group designated as ‘Total’ PD) with the rest. Patients were informed about their current periodontal health state. If severe PD was apparent, the patient received an additional information letter with encouragement to visit the dentist.

### 2.4. Statistical Analysis

IBM SPSS version 28.0 (IBM Corp, Armonk, NY, USA) was used to analyse the data. Chi-square tests were used to examine the possible differences of the distribution of PD groups among the patient groups (with/without multimorbidity) and disease chapters. Odds ratios (OR) and 95% confidence intervals (95% Cl) were calculated. A significance level of <0.05 was applied.

## 3. Results

### 3.1. Population Characteristics

Overall, 345 patients were included in this study. The characteristics of the study population are presented in Table 1. The mean age of the total population was 55.1 years (SD 16.0; range from 18 to 90); 54.2% were males and 15.4% of the patients were current smokers. The majority of the population was from Caucasian origin (72.5%), with no diploma beyond high school (66.4%). The BMI for the participants was 26.8 ± 11.3 kg/m^2^.

### 3.2. Diseases and Comorbidities

In total, 50 different diseases were extracted from the medical records representing 17 disease chapters after categorization using the 2022 MeSH terms (Table 2). It appeared that 15 patients had consulted the internist for evaluation; however, no specific diagnosis was established. The most common diseases were hypertension (22.6%), hypercholesterolemia (22.0%), DM2 (15.4%) and human immunodeficiency virus (HIV) infection (11.6%). The most common disease chapters were cardiovascular diseases (CVD) (39.1%), nutritional and metabolic diseases (31.2%) and endocrine systemic diseases (24.3%), while development anomalies (0.6%), eye diseases (0.3%) and infections (0.3%) were rarely seen in the current study population (Table 2). Notably, although HIV and hepatitis are chronic infectious diseases, they are categorized into two separate disease chapters, namely, digestive system diseases and viruses, respectively (Table 2).

The number of subjects with multimorbidity are presented in Table 3. Of all patients, 165 (47.8%) patients had 0 or 1 disease, of which 15 (4.3%) had no diagnosed disease and 150 individuals (43.5%) had one disease. A total of 82 (23.8%) patients presented with two systemic conditions, whereas 52 (15.1%) suffered from three systemic conditions and 45 (13.3%) from 4 or more diseases (Table 3). Overall, 180 patients (52.2%) suffered from multimorbidity (i.e., from two or more diseases).

### 3.3. Prevalence of Periodontal Disease and Association with Other Diseases

Next, we explored the prevalence of PD within the total study group. There were 56 (16.2%) individuals suffering from severe PD, while 185 (53.6%) suffered from severe or mild PD (i.e., Total PD). When considering severe PD among participants with zero to one disease or with multimorbidity, the prevalence was 44.6% and 53.6%, respectively. Corresponding frequencies for Total PD were 47.6% and 52.4%, respectively (Table 3). 

In Table 4, we present the prevalence of severe PD and Total PD distributed over the different disease chapters. When considering the disease chapters with at least 10 subjects, the prevalence of severe PD is the highest in disease chapter CVD (22.2%), urogenital diseases (21.0%) and nutritional and metabolic diseases (18.9%), while nervous system diseases (61.1%), CVD (60.7%) and digestive system diseases (58.3%) were the most common disease chapters in those with severe or mild (Total) PD. 

For each individual disease chapter, the risk of having severe or Total PD was calculated by means of OR. Only cardiovascular diseases were found to be significantly associated with severe PD (OR: 2.33 (95% CI; 1.25 to 4.33)) and with Total PD (OR: 1.61 (95% CI; 1.04 to 2.50)). A subsequent analysis took place to examine which of the twelve occurring cardiovascular diseases were significantly associated with PD status. It showed that myocardial infarction was significantly associated with severe PD (OR: 4.68 (95% CI; 1.27 to 17.25)).

### 3.4. Multimorbidity and Periodontal Status

To explore the association between multimorbidity and PD status, odds ratios and 95% CI were calculated. Suffering multimorbidity (having ≥2 diseases) did not clearly increase the risk of severe periodontitis (Table 5); the odds ratio for those suffering from ≥2 diseases versus ≤1 was 1.27 (95% CI; 0.69 to 2.34)). There was a similar result for patients suffering from ≥3 diseases versus ≤2 (OR: 1.65 (95% CI; 0.85 to 3.20)). The analyses for the associations between multimorbidity and total periodontitis yielded similar results (Table 5).

Among the total study participants, four diseases had a prevalence higher than 10%; these diseases were hypertension, hypercholesterolemia, diabetes mellitus type 2 and HIV (Table 2). We calculated the prevalence of PD for those suffering from any combination of two conditions (specific multimorbidity) (Table 6). The multimorbidity hypertension and DM2 (*n* = 18), followed by the multimorbidity hypertension and hypercholesterolemia (*n* = 15) and hypercholesterolemia and DM2 (*n* = 6), were shown to be the most prevalent disease combinations. It appeared that in all analysed multimorbidity combinations, PD (severe or Total PD) was shown to be highly prevalent.

## 4. Discussion

NCDs are a major health problem worldwide. The prevalence of NCDs is growing globally and their impact on the disease burden is considerable. The interplay of PD with certain NCDs has been portrayed extensively; several studies showed that periodontal-diseased patients suffer from an increased number of systemic conditions [28,35,36,37]. This current pilot study was initiated by including patients already suffering from a disease rather than having a PD case–control design; we assessed the prevalence of PD among individuals suffering from multimorbidity visiting the outpatient clinic of the Department of Internal Medicine in an academic hospital in The Netherlands. The data for the current secondary analyses came from the databases of two study cohorts. Severe and Total PD were more often present in individuals suffering from a higher number of systemic conditions (≥2, defined as multimorbidity), but this trend was not significant. However, of all the analysed disease chapters, cardiovascular diseases were shown to be significantly associated with severe PD and Total PD. 

So far, few studies investigated whether the number of systemic conditions in multimorbidity patients is associated with periodontal health status. One study conducted in Hong Kong did investigate whether periodontal health status reflects the concurrent presence of eight common systemic comorbidities using full-mouth periodontal examination, self-perceived health status questionnaires and 20 medical diagnostic tests [38]. It showed that those with worse periodontal conditions presented with poorer diagnostic test results and suffered from more systemic conditions. However, when comparing the comorbidity profiles with the severity of PD, most of them were not significant [38]. An Austrian study also indicated no association between the number of systemic conditions and the severity of PD [36]. These results are somewhat in line with the current results, since the trend of more PD among multimorbidity was not significant. We hypothesized that within a disease-affected cohort from an outpatient clinic of the Department of Internal Medicine, a worse periodontal health status could be found. Several factors could attribute to the current results, in that PD was highly prevalent in multimorbidity patients although not significant: First, the small study population; for example, a 10-fold increase and a multi-departmental and multicentre study design may show other results. This present study targeted a certain population by only recruiting from an outpatient clinic of internal medicine. On the other hand, the Hong Kong study did have a small patient cohort (*n* = 115)—three times smaller than this study (*n* = 345). However, an Austrian study, using a population cohort of 1119 patients, did not find an association between number of conditions and severity of PD [36,38] either. Secondly, the mean BMI in this study was 26.8 kg/m^2^ (±11.3), which, according to the World Health Organization, is not within the normal range (i.e., BMI 18.5 to 24.99 kg/m^2^) [39]. Pooled cohort studies showed that cardiometabolic multimorbidity (the co-existence of two or more of three cardiometabolic disorders: hypertension, diabetes mellitus and cardiovascular disease) increases as BMI increases; this could also be the case in some of the patients included [40]. It should be emphasized that dental health care in the Netherlands is relatively good, with an emphasis on quality, safety and the prevention of oral diseases [41]. Since we retrieved no dental health records, it is feasible that the recruited patient group did, in fact, have access to periodontal care and early intervention or that successful periodontal treatment prevented the progression of PD. This, in some cases, could have led to considerable gingival recession following treatment, resulting in shallow pocket depths; therefore, the CPITN screening may have categorized these patients as not suffering from mild or severe PD (underestimation of the prevalence of PD). In fact, a full-mouth clinical periodontal examination in the hospital outpatient clinic was not feasible; therefore, periodontal examination was performed at locations using the CPITN as an alternative. Nevertheless, the prevalence of severe PD in the current study was 16.2%, which is exactly the prevalence found previously in The Netherlands in 60,174 dental patients [13].

An important significant association found in this study was between the disease chapter CVD and PD status (severe and Total PD), in line with several other observational studies. Subsequent analysis showed that only myocardial infarction appeared to be significantly associated with severe PD (OR: 4.68 (95% CI; 1.27 to 17.25)). As observed in this study, these results are comparable to other studies in the past where the association between MI and PD was consistent across various PD definitions [42]. Furthermore, another study illustrated that those with acute myocardial infarction have worse PD status when compared to individuals without acute myocardial infarction [43]. PD has been suggested as a risk factor for the development of CVD due to systemic inflammation, and this current study adds again to this association [13,44,45].

Whether the PD found among the patients with morbidities at the outpatient clinic of internal medicine should be classified as recently proposed in the new PD classification system, is debatable [46]. They proposed PD in patients with a primary systemic disease should be grouped into the category “Systemic Diseases or Conditions Affecting the Periodontal Supporting Tissues” and not as the commonly occurring PD [30,46]. However, insufficient evidence is currently at hand to determine for each individual patient whether they suffered PD as a consequence of their systemic disease or whether they suffered simultaneously from their systemic disease and common occurring prevalent PD. We propose that PD can be part of a multimorbidity syndrome where the systemic disease could exacerbate PD but does not initiate PD. Interestingly, if we count severe PD as another condition (counting towards suffering from multimorbidity), the prevalence of multimorbidity in the total study population would increase from the currently reported 52.2% to 59.4%.

Multimorbidity is the presence of two or more chronic conditions in an individual where certain conditions can be causal to each other or share underlying disease mechanisms [5,6,7,8]. However, the cause of the combination of conditions remains largely unclear since certain potential relationships among these comorbidities are still unknown. Several studies showed that common systemic conditions, such as DM, rheumatoid arthritis and obesity, can be cofactors in the aggravation of periodontitis via different specific systemic pathways [21,30,47,48,49]. On the other hand, PD could also play a role in the exacerbation of existing or new conditions. Studies already showed possible biological pathways underlying the association for the onset or exacerbation of certain systemic conditions, such as ACVD, Alzheimer’s disease and Inflammatory Bowel Disease (IBD) [15,28,50,51]. In addition, one study did show a significant association of periodontal status with the risk for onset of common systemic conditions. A substantially worse periodontal status was also determined in those who developed more comorbidities over an 18-year period [52]. However, to what extent multimorbidity plays a role in the exacerbation of severe PD or whether PD plays a systemic role in multimorbid patients remains unclear. It is known that PD and NCDs share common risk factors such as lifestyle factors, socio-environmental factors, genes and epigenetics [53,54,55]. These risk factors could also possibly play a considerable role in the exacerbation of PD or a systemic condition in those suffering from multimorbidity [56,57,58,59]. 

The research found certain multimorbidity clusters affecting specific organs or systems in the body and perhaps it is possible that a certain combination of systemic conditions is also associated with periodontitis [60]. The value to know to what extent PD affects overall health and what role it plays in multimorbidity patients is an important step toward identifying opportunities for targeted, patient-centred care, possibly including PD as a marker. To analyse the prevalence and risk of PD and to explore to what extent PD is a risk factor for the onset of conditions in multimorbidity patients, further research using a larger multimorbidity cohort is highly recommended. Cluster data analyses to assess whether a certain combination of conditions in multimorbidity patients are associated with periodontitis is also something to consider. 

In general, medical specialists need to be aware of the periodontal status of their patients, not only at the outpatient clinic. One particular study showed that the presence of gingival index, plaque index and gingival inflammation is high among hospitalized patients across various wards, including the internal medicine infirmary [61]. In addition, limited knowledge of using dental aids within this group was observed [61]. Oral health will improve through dental and oral hygienist visits, also by stimulating the use of correct therapeutics; for example, using an electric toothbrush, fluoridated toothpaste and interdental aids. Further, as some studies suggested, possibly the use of natural substances, such as probiotics, could improve the state of eubiosis of the oral cavity [62,63,64,65].

## 5. Conclusions

This cross-sectional pilot study is one of the first studies assessing the periodontal status of registered multimorbidity patients from an outpatient clinic of internal medicine. It provides evidence to what extent PD is present in individuals suffering from multimorbidity. According to the present study, no statistically significant association between the number of diseases and PD status was discovered. However, PD prevalence is significantly increased in the CVD disease chapter and among those with a history of nonfatal myocardial infarction. A non-significant, increased risk was found for severe and Total PD in those suffering from multimorbidity. The conclusion remains unclear as to whether the multimorbidity played a role in the onset and progression of (severe) PD and whether PD plays a systemic role in multimorbid patients. More, larger epidemiological studies are needed among multimorbid patients. Furthermore, proactive action, such as screening for PD by medical specialists, is recommended; this in conjunction with the self-reported questionnaire to screen for PD (from Verhulst et al. (2019) [66] and Nijland et al. (2021) [31]) to refer the patients to an oral health specialist if necessary and introducing oral health prevention programs in hospitals [61]. 

## Figures and Tables

**Table 1:** Characteristics of the study population.

	Total Study Population (*n* = 345)
Age (years)	55.1 ± 16.0
Sex	
Female	158 (45.8)
Male	187 (54.2)
Ethnicity	
Caucasian	250 (72.5)
Non-Caucasian	95 (27.5)
Education	
≤High school	229 (66.4)
>High school	116 (33.6)
Smoking (current)	
Current	53 (15.4)
None	292 (84.6)
BMI (kg/m^2^)	26.8 ± 11.3

Values represent mean ± SD or *n* (%); BMI, body mass index; SD, standard deviation.

**Table 2:** Distribution of patients over the various disease chapters and specified systemic diseases.

Disease Chapter	*n* (%)	Systemic Diseases	*n* (%)
Infections	1 (0.3%)	Lyme disease	3 (0.9)
Neoplasm	28 (8.1%)	Cancer	28 (8.1)
Musculoskeletal diseases	24 (7%)	Arthritis	15 (4.3)
		Other bone diseases	14 (4.1)
Digestive system diseases	36 (10.4%)	Liver disease	7 (2.0)
		IBD (Crohn’s disease and colitis ulcerosa)	5 (1.4)
		Gastroesophageal reflux disease	1 (0.3)
		Hepatitis A, B and C (Liver diseases)	3 (0.9)
		Other digestive system conditions	17 (4.9)
		Pancreatitis	4 (1.2)
Respiratory tract diseases	18 (5.2%)	Hyperventilation	1 (0.3)
		Asthma	4 (1.2)
		Chronic Bronchitis and pulmonary emphysema	5 (1.4)
		Lung diseases	9 (2.6)
Nervous system diseases	36 (10.4%)	Brain infarction	13 (3.8)
		Migraine	5 (1.4)
		Peripheral nervous system diseases	8 (2.3)
		Migraine	5 (1.4)
Eye diseases	1 (0.3%)	Glaucoma	7 (2.0)
Urogenital diseases	19 (5.5%)	Kidney disease	18 (5.2)
		Prostatitis	1 (0.3)
Cardiovascular diseases	135 (39.1%)	Hypertension	78 (22.6)
		Angina pectoris	16 (4.6)
		Myocardial infarction	13 (3.8)
		Angioedema	3 (0.9)
		Heart failure	7 (2.0)
		Cardiac arrhythmias	17 (4.9)
		Pericarditis	4 (1.2)
		Aortic valve stenosis	3 (0.9)
		Cardiomyopathies	2 (0.6)
		Peripheral arterial disease	12 (3.5)
		Atherosclerosis	7 (2.0)
		Embolism and Thrombosis	32 (9.3)
Hemic and lymphatic	35 (10.1%)	Thrombocythemia	10 (2.9%)
diseases		Lymphedema	3 (0.9%)
Skin and connective tissue diseases	16 (4.6%)	Skin conditions	16 (4.6)
Nutritional and metabolic diseases	111 (32.2%)	Hypercholesterolemia	76 (22.0)
		Dyslipidaemias	21 (6.1)
		Hypertriglyceridemia	3 (0.9)
		Hyperlipidaemias	16 (4.6)
Endocrine systemic diseases	84 (24.3%)	Diabetes mellitus Type 1 (DM1)	10 (2.9)
		Diabetes mellitus Type 2 (DM2)	53 (15.4)
		Hyperthyroidism	12 (3.5)
		Hypothyroidism	12 (3.5)
Immune system diseases	8 (2.3%)	Rheumatoid arthritis	13 (3.8)
		Sjogren Syndrome	1 (0.3)
		Other auto-immune conditions	7 (2.0)
Mental disorders	11 (3.2%)	Psychiatric disorders	11 (3.2)
Viruses	40 (11.6%)	HIV	40 (11.6)
Development anomalies	2 (0.6%)	Asplenia	2 (0.6)

Categorization of each extracted disease by disease chapter using the 2022 Medical Subject Headings thesaurus (MeSH) from the U.S. National Library of Medicine. Values represent *n* (%); note, values add up to higher numbers (%s) than the total study population due to multimorbidity. For 15 patients, no disease was diagnosed. HIV, human immunodeficiency virus.

**Table 3:** Prevalence of multimorbidity in the total study population.

Multimorbidity	Total Study Population *n* = 345	Severe Periodontal Disease *n* = 56	Total Periodontal Disease *n* = 185
0 and 1 disease	165 (47.8)	25 (44.6)	88 (47.6)
2 diseases	82 (23.8)	12 (21.4)	38 (20.5)
3 diseases	52 (15.1)	11 (19.6)	31 (16.8)
≥4 diseases	46 (13.3)	8 (14.3)	28 (15.1)
Multimorbidity	180 (52.2)	30 (53.6)	97 (52.4)

Values represent *n* (%). Multimorbidity is defined as having two or more diseases.

**Table 4:** Prevalence of each disease chapter and its associations with the periodontal status.

Disease Chapters	Severe Periodontal Disease *n* (%)	Severe Periodontal Disease OR (95% CI)	Total Periodontal Disease *n* (%)	Total Periodontal Disease OR (95% CI)
Cardiovascular diseases (*n* = 135)	30 (22.2)	2.33 (1.25 to 4.33)	82 (60.7)	1.61 (1.04 to 2.50)
Nutritional and metabolic diseases (*n* = 111)	21 (18.9)	1.25 (0.66 to 2.35)	59 (53.2)	0.97 (0.62 to 1.53)
Endocrine systemic diseases (*n* = 84)	15 (17.9)	1.14 (0.57 to 2.27)	45 (53.6)	1.00 (0.61 to1.63)
Viruses (*n* = 40)	5 (12.5)	0.73 (0.26 to 2.65)	21 (52.5)	0.95 (0.49 to 1.84)
Nervous system diseases (*n* = 36)	4 (11.1)	0.80 (0.25 to 2.55)	22 (61.1)	1.41 (0.70 to 2.85)
Digestive system diseases (*n* = 36)	5 (13.9)	0.95 (0.33 to 2.74)	21 (58.3)	1.24 (0.62 to 2.49)
Hemic and lymphatic diseases (*n* = 35)	4 (11.4)	0.51 (0.11 to 1.55)	14 (40.0)	0.54 (0.27 to 1.11)
Neoplasm (*n* = 28)	5 (17.9)	1.02 (0.35 to 2.98)	14 (50.0)	0.85 (0.39 to 1.85)
Musculoskeletal diseases (*n* = 24)	12 (12.5)	0.70 (0.19 to 2.57)	12 (50.0)	0.86 (0.37 to 1.96)
Respiratory tract diseases (*n* = 18)	3 (16.7)	0.76 (0.21 to 2.85)	7 (38.9)	0.53 (0.20 to 1.41)
Urogenital diseases (*n* = 19)	4 (21.0)	1.29 (0.38 to 4.37)	10 (52.6)	0.96 (0.38 to 2.42)
Skin and connective tissue diseases (*n* = 16)	1 (6.3)	0.35 (0.04 to 2.83)	8 (50.0)	0.86 (0.32 to 2.34)
Mental disorders (*n* = 11)	0 (0.0)	0.73 (0.67 to 0.80)	5 (45.5)	0.71 (0.21 to 2.38)
Immune system diseases (*n* = 8)	0 (0.0)	0.56 (0.06 to 4.93)	0 (0.0)	0.51 (0.12 to 2.17)
Development anomalies (*n* = 2)	N/A	N/A	N/A	N/A
Eye diseases (*n* = 1)	N/A	N/A	N/A	N/A
Infections (*n* = 1)	N/A	N/A	N/A	N/A

Values represent *n* (%) or odds ratios (OR) and corresponding 95% confidence intervals (CI); N/A, not applicable. Note, values for *n* (%) add up to higher numbers than the total study population due to multimorbidity.

**Table 5:** Multimorbidity in association with the periodontal disease status.

	0 and 1 vs. ≥2 Diseases	0, 1 and 2 vs. ≥3 Diseases
	OR (95% Cl)	OR (95% Cl)
Severe periodontal disease	1.27 (0.69 to 2.34)	1.65 (0.85 to 3.20)
Total periodontal disease	1.23 (0.81 to 1.88)	1.54 (0.96 to 2.48)

Values represent odds ratios (OR) and corresponding 95% confidence intervals (CI). vs., versus.

**Table 6:** Prevalence of multimorbidity combinations for the most prevalent diseases.

Specific Multimorbidity: Combinations of the Most Prevalent Diseases	Severe Periodontal Disease *n* = 56	Prevalence of Severe Periodontal Disease in Multimorbidity Combination	Total Periodontal Disease *n* = 185	Prevalence of Total Periodontal Disease in Multimorbidity Combination
Hypertension and DM2 (*n* = 18)	4 (7.1%)	22.2%	12 (6.5%)	66.7%
Hypertension and Hypercholesterolemia (*n* = 15)	3 (5.5%)	20.0%	6 (3.2%)	40.0%
Hypercholesterolemia and DM2 (*n* = 6)	1 (1.8%)	16.7%	2 (1.1%)	33.3%
Hypertension and HIV (*n* = 4)	2 (3.6%)	50.0%	4 (2.2%)	100%
Hypercholesterolemia and HIV (*n* = 1)	1 (1.8%)	100%	1 (0.5%)	100%
DM2 and HIV (*n* = 1)	0 (0.0%)	0.0%	1 (0.5%)	100%

Values represent *n* (%), the prevalence of multimorbidity combination in the PD status groups. DM2, diabetes mellitus type 2; HIV, human immunodeficiency virus.

## Data Availability

The data presented in this study are available on request from the corresponding author.
